# Man the Fat Hunter: The Demise of *Homo erectus* and the Emergence of a New Hominin Lineage in the Middle Pleistocene (ca. 400 kyr) Levant

**DOI:** 10.1371/journal.pone.0028689

**Published:** 2011-12-09

**Authors:** Miki Ben-Dor, Avi Gopher, Israel Hershkovitz, Ran Barkai

**Affiliations:** 1 Institute of Archaeology, Tel Aviv University, Tel Aviv, Israel; 2 Department of Anatomy and Anthropology, Sackler Faculty of Medicine, Dan David Laboratory for the Search and Study of Modern Humans, Tel Aviv University, Tel Aviv, Israel; Illinois State University, United States of America

## Abstract

The worldwide association of *H. erectus* with elephants is well documented and so is the preference of humans for fat as a source of energy. We show that rather than a matter of preference, *H. erectus* in the Levant was dependent on both elephants and fat for his survival. The disappearance of elephants from the Levant some 400 kyr ago coincides with the appearance of a new and innovative local cultural complex – the Levantine Acheulo-Yabrudian and, as is evident from teeth recently found in the Acheulo-Yabrudian 400-200 kyr site of Qesem Cave, the replacement of *H. erectus* by a new hominin. We employ a bio-energetic model to present a hypothesis that the disappearance of the elephants, which created a need to hunt an increased number of smaller and faster animals while maintaining an adequate fat content in the diet, was the evolutionary drive behind the emergence of the lighter, more agile, and cognitively capable hominins. Qesem Cave thus provides a rare opportunity to study the mechanisms that underlie the emergence of our post-erectus ancestors, the fat hunters.

## Introduction

It is our contention that two distinct elements combined in the Levant to propel the evolutionary process of replacing *H. erectus* by a new hominin lineage ([1], As the classification of varieties of the genus *Homo* is problematic, we refrain in this paper from any taxonomic designations that would indicate species or subspecies affiliation for the hominins of Qesem Cave. The Qesem Cave hominin, based on the analysis of teeth shares dental characteristics with the Skhul/Qafzeh Middle Paleolithic populations and to some extent also with Neandertals). One was the disappearance of the elephant (*Elephas antiquus*) – an ideal food-package in terms of fat and protein content throughout the year – which was until then a main calorie contributor to the diet of the *H. erectus* in the Levant. The second was the continuous necessity of *H. erectus* to consume animal fat as part of their diet, especially when taking into account their large brains [2]. The need to consume animal fat is the result of the physiological ceiling on the consumption of protein and plant foods. The obligatory nature of animal fat consumption turned the alleged large prey preference [3], [4] of *H. erectus* into a large prey dependence. Daily energy expenditure (DEE) of the hominins would have increased when very large animals such as the elephant had diminished and a larger number of smaller, faster animals had to be captured to provide the same amount of calories and required fat. This fitness pressure would have been considerably more acute during the dry seasons that prevail in the Levant. Such an eventuality, we suggest, led to the evolution of a better equipped species, in comparison with *H. erectus*, that also had a lighter body [5], a greater lower limb to weight ratio ([6]:194), and improved levels of knowledge, skill, and coordination ([7]:63) allowing it to better handle the hunting of an increased number of smaller animals and most probably also develop a new supporting social organization.

We also suggest that this evolutionary process was related to the appearance of a new and innovative local cultural complex – the Levantine Acheulo-Yabrudian [8], [9]. Moreover, a recent study of dental remains from the Acheulo-Yabrudian site of Qesem Cave in Israel dated to 400-200 kyr ago [10], [11] has indicated that the hominins inhabiting the cave were not *H. erectus* but were rather most similar to later populations (e.g., Skhul/Qafzeh) of this region ([1] and references therein).

### The Broader Context

Our direct ancestor, *H. erectus*, was equipped with a thick and large skull, a large brain (900 cc on average), impressive brow ridges and a strong and heavy body, heavier than that of its *H. sapiens* successor (e.g., [12], [13], [14]). Inhabiting the old world for some 1.5 million years, *H. erectus* is commonly associated with the Acheulian cultural complex, which is characterized by the production of large flakes and handaxes – large tools shaped by bifacial flaking. Handaxes are interpreted as tools associated with the butchering of large game (e.g., [15], [16]). *H. erectus* was also suggested in recent years to have used fire [17], [18]; however the supporting evidence is inconclusive. Albeit the positive archaeological evidence from the site of Gesher Benot Ya'aqov (henceforth GBY) dated to around 780 kyr [19], [20], [21], the habitual use of fire became widely spread only after 400 kyr [22], [23], [24], [25].

Archaeological evidence seems to associate *H. erectus* with large and medium-sized game {Namely, Body Size Group A (BSGA Elephant, >1000 kg), BSGB (Hippopotamus, rhinoceros approx. 1000 kg), and BSGC (Giant deer, red deer, boar, bovine, 80–250 kg); (after [26])}, most conspicuously elephants, whose remains are commonly found at Acheulian sites throughout Africa, Asia, and Europe (e.g., [26], [27], [28], [29], [30]). In some instances elephant bones and tusks were also transformed into shaped tools, specifically artifacts reminiscent of the characteristic Acheulian stone handaxes [31].

In Africa, *H. sapiens* appears around 200 kyr ago, most probably replacing *H. erectus* and/or *H. heidelbergensis* [32], [33], [34]. Early African *H. sapiens* used both handaxes and the sophisticated tool-manufacturing technologies known as the Levallois technique (e.g., [35], [36]) while its sites are devoid of elephants [32], [35]. The presence of elephants in many Acheulian African sites and their absence from later Middle Stone Age sites [29], [37], evoked an overkill hypothesis ([38]:382), which was never convincingly demonstrated. Thus no link was proposed, in the case of Africa, between human evolution and the exclusion of elephants from the human diet, and no evolutionary reasoning was offered for the emergence of *H. sapiens* in Africa [39].

In Europe, *H. erectus* was replaced by *H. heidelbergensis* [40] and later by hominins associated with the *Neanderthal* evolutionary lineage [41]. In spite of significant cultural changes, such as the adoption of the Levallois technique and the common use of fire, the manufacture and use of handaxes and the association with large game persisted in post-*erectus* Europe until the demise of the *Neandertals*, around 30 kyr BP (e.g., [42]). *H. sapiens* did not evolve in Europe but migrated to it no earlier than 40 kyr BP (e.g., [43]).

In the Levant, dental remains from the Acheulo-Yabrudian site of Qesem Cave, Israel [10], [11] demonstrate resemblance to dental records of later, Middle Paleolithic populations in the region [1] indicating that *H. erectus* was replaced some 400 kyr ago by a new hominin ancestral to later populations in the Levant. A rich and well-dated (400-200 kyr) archaeological dataset known from the Levant offers a glimpse into this significant process and a better understanding of the circumstances leading to the later emergence of modern humans thus suggesting a possible link between the cultural and biological processes. This dataset pertains to the unique local cultural complex known as the Acheulo-Yabrudian, a diversified and innovative cultural complex (e.g., [8], [44], [45]), which appeared some 400 kyr ago, immediately following the Acheulian cultural complex [10], [11], and which lasted some 200 kyr. Acheulo-Yabrudian sites as well as sites associated with subsequent cultures in the Levant show no elephant remains in their faunal assemblages.

## Materials and Methods

The Model and its Components: The bioenergetic model we offer combines two sets of data. The first concerns the notion of obligatory animal fat consumption as found in the literature and our own calculations. The second concerns the role of elephants in human diet and a comparison of faunal data from two Middle Pleistocene, Acheulian and Acheulo-Yabrudian sites in the Levant.

### The Obligatory Animal Fat Dietary Model

The ongoing increase in human brain size{Average human encephalization quotient (actual/expected brain mass for body weight) is slightly over 6.0, compared to values between 2.0 and 3.5 for hominids and primates [46]} had its toll: it became the most metabolically energy-expensive organ in the human body, consuming 20–25% of the adult and 70–75% of the newborn metabolic budget [47]. In order to not exceed the human limited “energy budget” (dictated by basal metabolic rate), shrinkage in gut size (another metabolically energy expensive organ) was a necessary accompaniment. It was Aiello and Wheeler [2] who suggested that gut size was a constraining factor on potential brain size, and vice versa. A shorter human gut, henceforth, had evolved to be more dependent on nutrient and energy-dense foods than other primates. The more compact, the human gut is less efficient at extracting sufficient energy and nutrition from fibrous foods and considerably more dependent on higher-density, higher bio-available foods that require less energy for their digestion per unit of energy/nutrition released. It would therefore appear that it was the human carnivorousness rather than herbivorous nature that most probably energized the process of encephalization throughout most of human history [2], [48], .

#### The physiological ceiling on protein intake

It is known that diets deriving more than 50% of the calories from lean protein can lead to a negative energy balance, the so-called “rabbit starvation” due to the high metabolic costs of protein digestion [51], [52], as well as a physiological maximum capacity of the liver for urea synthesis [53], [54]. The conversion of protein to energy requires liver enzymes to dispose of nitrogen, an ingredient of amino acids which compose the protein molecule. Consumption of protein by humans is thus limited by the capacity of the liver to produce such enzymes and of the kidney to dispose of the urea – the nitrogen containing by-product from metabolism of proteins by the liver.

Several upper limits to the daily consumption of protein have been suggested:

High-protein weight-loss dieters who are physically very active were advised to consume protein up to 1.7 gr/kg bodyweight/day [55]. A dieter weighing 100 kgs should thus consume up to 170 grams of protein a day, which in terms of lean beef translates to approximately 566 grams ([56] after USDA SR-21) due to the large content of water in meat.

A recent review of the literature ([57]:888) recommends a long-term maximum protein intake of 2 gr/kg body weight/day.

Studying the maximum rate of urinary urea nitrogen excretion as well as the maximum rate of urea synthesis in men, Rudmanet al. ([58]:2246) concluded that a healthy person weighing 50 kg would convert a maximum of 190 g amino acids (protein) translated to a short term maximal protein consumption of 3.8 gr/kg bodyweight/day.

Both Cordain ([53]:688) and Speth ([4]:77) used a value of 35% of the total caloric intake as a maximum for protein intake, following Rudman's findings.

We have chosen to use Cordain and Speth's 35% protein calories ceiling showing (based on our assumptions in Table 1 and Table 2 regarding DEE and body weight) a 4.0 gr/kg body weight/day for *H. sapiens*. It should be emphasized however that the physiological ceiling on protein intake is not a percentage of total calories but rather a fixed quantity of calories so that with an increase in calorie intake beyond normal consumption and given a maximal intake of protein, the percentage of maximal calories from protein is reduced while consumption of either fat or plant foods is increased to achieve the total caloric requirements. It is possible to extrapolate a ceiling on protein intake for *H. erectus* from modern populations since little difference is evident between primates and modern man in their relative size of liver ([2]:205). Based on our assumptions (Table 1 and Table 2) for body weight and DEE for *H. erectus*, his ceiling works out to be 3.9 gr/kg body weight/day.

**Table 1:** Hominin weight and MQ estimates ([5]:258; in bold: the species on which we elaborate).

	Brain weight (g)	Male weight (kg)	Female weight (kg)	Post-canine surface (mm^2^)	Megadontia quotient (MQ)
A. afarensis	438	45	29	460	1.7
A. africanus	452	41	30	516	2.0
A. boisei	521	49	34	756	2.7
A. robustus	530	40	32	588	2.2
**H. habilis**	**601**	**37**	**32**	**478**	**1.9**
H. rudolfensis	752	60	51	572	1.5
H. ergaster	849	66	56	377	0.9
**H. erectus**	**1019**	**66**	**56**	**402**	**1.0**
H. heidlbergensis	1200	77	56	389	0.9
H. neanderthalensis	1362	77	66	335	**0.7**
**H. sapiens**	**1350**	**58**	**49**	**334**	**0.9**

**Table 2:** Obligatory requirements of animal fat at different DEE levels.

DEE^1^ cal.	Raw plant cal.^2^	Animal protein cal.^3^	Animal fat cal.^4^	% fat of total^5^	% fat of animal cal.^6^
**H. erectus**					
2704	1014	947	743	27%	44%
3500	1014	947	1539	44%	62%
**H. sapiens**					
2451	776	858	817	33%	49%

Table Notes.

1 DEE – Daily Energy Expenditure. After Leonard and Robertson ([156]:275) adjusted for average gender controlled weight per McHenry ([5]:258; and see and Table S1).

2 37.5% and 31.7% of DEE for *H. erectus* and *H. sapiens* respectively, based on equation above.

3 After Cordain et al. ([53]), based on Rudman et al. ([58]) and adjusted for body weight.

4 DEE minus calories supplied by consumption of animal protein and plant sources.

5 Animal fat calories divided by total calories obtained from all food sources.

6 Animal fat calories divided by total calories obtained from animal sources alone.

#### The physiological ceiling on plant food intake

The intake of plant foods can conceivably be limited due to a physiological ceiling on fiber or toxins intake, limited availability, or technological and time limitations with respect to required pre-consumption preparations, or a combination of these three factors. A significant contribution to the understanding of the physiological consequences of consuming a raw, largely plant-based diet was made by Wrangham et al. [17], [18]. A physiological limitation seems to be indicated by the poor health status of present-day dieters who base their nutrition on raw foods, manifested in sub-fecundity and amenorrhea [17], [18]. Presumably this limitation would have been markedly more acute if pre-agriculture highly fibrous plant foods were to be consumed. Limited availability is manifested by the travails of the obligated high-quality diet consumer of the savanna, the baboon. Baboons are somewhat similar to humans with respect to their ratio of colon to small intestine rendering the quality requirements of their diet comparable to an extent to that of humans. Baboons have been documented at times as devoting “almost all of their daylight hours to painstakingly seeking out small, nutritious food items….[the] adult male baboon (*Papio cynocephalus*) may pick up as many as 3000 individual food items in a single day” ([59]:103).

Nuts, or other high-quality foods of decent size appear only seasonally above ground in the savanna and such is the case in the Levant, too. But not only are they seasonal, they also require laborious collection and most of them contain phytic acid that inhibits the absorption of contained minerals. These foods also contain anti-nutrients and toxins such as trypsin, amylase and protease inhibitors as well as tannins, oxalate, and alkaloids the elimination of which can only be achieved (sometimes only partially) by pre-consumption processing like drying, soaking, sprouting, pounding, roasting, baking, boiling and fermentation. While these technologies are extensively used, mostly conjointly, in present day pre-consumption preparation of many plant foods, some were probably not practiced by *H. erectus*, especially those requiring accumulation and storage of produce for weeks (see, for example, [60]:173 with respect to Mongongo nuts, or [61]:31 regarding Baobab seeds). Comparing food class foraging returns among recent foragers, Stiner ([62]:160) has found the net energy yield of 3,520–6,508 kj/hour for seeds and nuts compared to 63,398 kj/hour for large game. Roots and tubers returns are not better, ranging from 1,882 kj/hour to 6,120 kj/hour. These numbers point to the substantial time investment required in gathering and preparing plant foods for consumption.

Some researchers (e.g., [63], [64]) suggested significant consumption of underground storage organs (USOs), such as tubers and roots that are not easily accessible to animals, as a possible source of high-quality plant food for early hominins in the savanna. Conklin-Brittain et al. [65] used fiber content as the principle measure defining a diet's quality. Fibers, they claim, displace nutritious elements in limited food digestive capabilities while also trapping additional nutrients within their matrix. Consequently and in view of their relatively low fiber content (16% of dry matter compared to 34% for fruit, 46% for seeds and 44% for pith), these researchers propose significant consumption of USOs by the australopithecines. In their view, “post-canine megadondtia and low rounded thick enameled cusps are all compatible with the physical challenges imposed upon dentition by the consumption of USOs … robust gnathic architecture … [is also compatible] in keeping with the heavy chewing that would have been required to comminute USOs” ([65]:66). None of these features were preserved in *H. erectus* [66]. In other words, *H. erectus* was not well equipped to consume USOs, even as a fallback diet. Moreover, analysis of *H. erectus* cranial features in comparison to those of *H. habilis*, shows gracilization of the jaws, increased occlusal relief, and reduction in post-canine tooth size indicating a continued emphasis on the shearing of food items during mastication and a reduction in the hardness of foods consumed [67], [68]. Dental analysis thus led Ungar ([69]:617) to determine that “meat seems more likely to have been a key tough-food for early *Homo* than would have USOs”.

It thus seems that the attractiveness of USOs as a source of high-quality nutrients would have been limited and that USO processing capabilities were diminished with the apparent evolution of the early *Homo* cranial and gut features. One way to handle this dietary challenge, as proposed by Wrangham et al. [17], [18] would have been by cooking. However, with respect to early *Homo*, this is not a likely suggestion and while some have recently claimed that the habitual use of fire emerged only some 400 kyr ago [22], [23], [24], [25], [70] other very recent statements argued in favor of an even later adoption of fire [71].

However, even where the habitual use of fire is known to have existed, it does not seem to lead to high consumption of plant-based foods among Paleolithic populations. Nitrogen (δ15N) isotope studies of *H. sapiens* in Upper Paleolithic Europe, where control of fire is clearly evidenced, demonstrate a diet rich in animal content rather than vegetal foods [72], [73], even in relatively temperate climates boasting extensive vegetation, such as France, Italy, Romania, and Croatia ([74]:252). Notwithstanding, isotope studies may underestimate consumption of plant foods low in protein such as tubers. Similarly, modern hunter-gatherer (HG) groups, despite having access to fire and metal tools, also seem to have a strong preference for carnivorous foods over vegetal foods ([53]:682), a notion also supported by a recent study [75] that emphasizes limited consumption of carbohydrates by present day HG groups.

Indeed, an analysis of nine HG groups for which detailed dietary information exists ([76]:166) shows that five groups, located in an area abundant in vegetation, consumed only a meager amount of plant foods (17% of calories on average). Availability of fatty plant foods like Mongongo nuts, Palm oil, and Baobab seeds is associated with the groups that exhibit high vegetal consumption. Speth ([4]:87–107) describes the preference of several present-day African tribes to hunt and exclusively consume large animals even in the midst of the most nourishing vegetal season. Speth attributes this tendency to cultural or even political motives, an interpretation with which we do not concur (see also [50]).

Recent support for the insignificant role of USOs in Paleolithic human diet may be derived from genetic data of present populations. Hancock et al. ([77]:8926) report that the strongest signals of recent genetic adaptations in the human genome of modern populations heavily dependent on roots and tubers, were to starch, sucrose, low folic acid contribution, and the detoxification of plant glycosides (found in tubers). The need for such an adaptation to so many specific USO attributes implies that humans were not previously adapted to consume significant quantities of USOs or any other form of dense starches for that matter. Similarly, Perry [78] reports that an increase in copies of salivary amylase gene (AMY1) begun to appear in humans probably only within the past 200 kyr. Needed to convert starch into glucose, chimpanzees have only two copies of amylase while present day humans have two to sixteen copies, indicating that adaptation to high starch consumption was probably not in place during the Middle Pleistocene (and even, as Perry discovered, is still not present in certain present day low starch consuming populations).

Another organ that should be examined when gauging the evolutionary route of humans with respect to fiber is the gut. Unlike humans, all herbivores have the ability to convert large quantities of vegetable fiber and other carbohydrates into short-chain fatty acids which they are able to absorb. Microfloral activity (i.e., fermentation) within their gastrointestinal tract ferment fibers and cellulose to produce these short-chain fatty acids. In fact, the natural diet of mammals is a high-fat diet. Therefore, any evidence of disengagement from fiber consumption would manifest itself in the colon. In weight, the human gut is about 60% of that which would be expected in a primate of similar size. This compensatory reduction, allowing for an increase in brain size while maintaining the necessary metabolic rate, stands against the notion of increased fiber consumption ([2]:204) in as much as the reduced weight is mostly attributed to a very short colon ([59]:99), which in *H. sapiens* comprises only 20% of their (relatively smaller) gut. In comparison, the chimpanzee's (*Pan troglodytes*) colon comprises 52% of its gut. Aiello and Wheeler ([2]:210) infer that African *H. erectus* also had a relatively small gut based on the proportions of their thoracic cage and pelvis which are similar to those of *H. sapiens*.

#### Using teeth data to gauge the physiological ceiling on plant consumption

The need for an evolution of a coordinated digestive system, including both the teeth and the gut is described by Lucas et al. ([79]:35). Acting in concert, a coordinated effort is required to ensure flow from the molars to the gut that would not translate to an avalanche of fibrous food. These researchers' note, in regard to the evolutionary responsiveness of the teeth's shape and size, that the use of “any part of the body for 3000 times a day is unlikely to escape selection pressures” ([79]:39).

To estimate the physiological limitation placed on the digestion of raw fibrous foods, we thus use McHenry's megadontia quotient, or MQ [5], [67]. MQ takes into account postcanine tooth area in relation to body mass. McHenry himself states that the MQ index was not meant to be precise, however it offers a noted sensitivity, ranging from 0.7 to 2.7 (see Table 1), and showing distinguished values of 0.9 for *H. sapiens* and 0.7 for the highly carnivorous *H. neanderthalensis* [73].

Reduction in teeth size can be attributed to either a change in diet or the use of exogenous food preparation techniques through the use of tools. It is therefore logical to start our comparison with the earliest tool-using hominin, the *H. habilis*. It is remarkable that the MQ (1.9) of *H. habilis* is closer to that of the Australopithecines and, by average, is nearly double that of the genus *Homo* as a whole. Given that *H. habilis* and *H. erectus* sought food in comparable savanna environments, the difference in diet, as seen from the point of view of molar size and topography, is quite extensive.

In order to get an initial estimate of a plant food ceiling we assumed a modest 10% animal diet for *H. habilis* (MQ = 1.9) and 80% animal diet for *H. neanderthalensis* (MQ = 0.7) as two extreme points. Assuming linear relation of MQ to Y – the percentage of plant food ceiling – allows the formation of a linear equation Y = 0.583MQ-0.208. The estimate for *H. erectus* (MQ-1.0) is thus 37.5%. We find this number to be slightly high in view of the HG record, the δ15N isotope studies, the genetics record, and a reasonable estimate of the physiological, inventory and time limitations for raw, non-cooked, plant foods such as USOs, nuts, and seeds. We have decided however to use this result in our following calculations, with the aim of maintaining strict assumptions.

For *H. erectus*, whose DEE is estimated at 2704 calories (see Table 2 and Table S1), we reach a maximum long-term plant protein ceiling of 1014 calories. This level of vegetal consumption means that *H. erectus* was indeed an omnivore whose diet was significantly varied (e.g., [80]).

#### The critical role of animal fat

If dietary consumption of animal protein and raw plant food is physiologically limited, how could *H. erectus* provide for his caloric requirements? This question was posed by Wrangham et al. [17], [18], underlying their “Cooking Hypothesis”. However, assuming that fire was not routinely available for cooking, we propose that the answer may lie in the obligatory consumption of animal fat. Documented long-term consumption of high fat diets in traditional societies shows no negative health effects, e.g., 66% fat in the Masai diet [81], or 48% up to 70% fat in the Inuit diet ([82]:652, [4]:76). Consumption of fat by early *Homo* was thus suggested as a possible solution to the “protein poisoning” problem, as the result of excessive protein intake is sometime referred to ([4]:337). In addition, a strong tendency to target fat was evidenced as early as 1.9 Ma ago by bone marrow processing as seen at the Olduvai and Koobi Fora African sites ([3]:371). In addition, the facts that meat proteins digestion is costlier compared to fat [83] and that a larger percentage of protein escapes digestion while fat digestion is nearly complete [84], [85] also render animal fat a very efficient energy source.

It is not often realized that fat forms a large part, and sometimes a major part, of most mammals' diet, including the great apes. Gorillas, for example, obtain about 60% of their energy from fat [86] produced through the fermentation of plant fiber by bacteria found in their large intestine. It is estimated that each gram of fiber is processed by bacteria to yield 1.5 calories in the form of short-chain saturated fatty acids that are absorbed into the blood stream through the large intestinal wall. It is precisely the large intestine, the fat-producing apparatus in the great apes, which is diminished in humans to offset the increased brain volume, creating a shortage of fat in human diets. Replacement by exogenous fat consumption from animal sources would have been the least metabolically demanding alternative. It is therefore not surprising that the most recent human genetic adaptations are to high starch consumption, which must have been the least prevalent component in the diet of our lineage during the Pleistocene.

With upper consumption ceilings of animal protein (947 calories) and plant foods (1014 calories), our model (Table 2), assuming DEE of 2704 for *H. erectus*, would require a daily intake of 744 calories originating in animal fat, comprising 27% of total calories and 44% of animal source calories.

### Elephants and animal fat in the Levant 800–200 kyr BP

In the absence of data regarding Levantine fauna's protein and fat content we use data for African fauna of similar weight as representatives of the relevant Levantine fauna found in GBY and Qesem Cave (Table 3 and Table S2).

**Table 3:** Caloric protein and fat contribution of representative animals (and see Table S1).

Animal	M/F	Liveweight^1^ (kg)	% fat liveweight^2^	Fat cal. (9 cal/g)^3^	Protein cal. (4 cal/g)^4^	% fat cal.
**Elephant (** ***Elephas antiquus*** **)** 5		Est.6,952	4.1%	2,117,322	2,182,603	49%
**Hipopotamus Amph.**	Both	1,383.0	5.0%	512,541	401,200	56%
**Buffalo**	M	753.0	4.1%	229,297	236,400	49%
**Oryx antelope**	Both	168.5	4.0%	49,151	68,000	42%
**Kob antelope**	Both	79.4	2.9%	17,048	38,400	31%
**Thomson's gazelle (N)**	Both	21.9	2.1%	3,395	10,000	25%
**Thomson's gazelle (S)**	Both	18.6	2.2%	3,026	7,200	30%

Table Notes.

1 Excluding brain, heart, liver, tongue together comprising approximately 5% of edible tissue by weight ([157]:1626).

2 All numbers for fat with the exception of the elephant are based on Bunn and Ezzo ([3]:370 after Ledger ([87]). Cancellous fat is excluded.

3 Fat yield = 82% of fat weight. Based on Cordain et al. ([88]:152) after Crawford et al. ([89]).

4 Protein content based on Ledger ([87]:299).

5 Elephant weight based on Christiansen ([158]: Table 7); fat to protein proportion based on buffalo here. Due to lack of data regarding fat and protein content of elephants, and ancients one at that, our calculations are based on the next largest terrestrial animal mentioned by Ledger ([87]) – the buffalo. As the proportion of fat increase with the size of the animal ([159]), the estimated fat content and total calories supplied by the elephant are likely underestimated.

Data in Table 3 were extracted from Table 1 in Bunn & Ezzo [3] which, in turn, are based on Ledger's [87] data of carcass and offal fat and a 2.4% estimate of marrow fat for male white-tailed deer. We did not include cancellous fat under the assumption that it would probably not have been extractable in the absence of cooking. All fat weights in kg were multiplied by 9,000 in order to obtain calories and multiplied by 0.82 to express partial fat yield as per Cordain et al. ([88]:152) after Crawford et al. [89]. Protein data were extracted from Ledger ([87]: Table I), and weights in kg were multiplied by 4,000 to obtain caloric values. Where available average values for males and females were considered. These data are further detailed in supporting materials.

Ledger [87] sampled only animals in “good conditions”, however, the method he employed tends to underestimate animal fat content as it ignores intramuscular fat. A chemical extraction method would provide higher values. For example, applying a chemical extraction method to the southern hairy-nosed wombat [90] whose natural habitat is semi-arid, yielded 49% calories from fat on average (from a total weight of 14–28 kgs) while slightly larger animals in our table (e.g., the Gazelle) yielded 30–36% calories from fat (allowing for the above mentioned 82% yield adjustment). We presume that the wide range indicated by the study between the minimum (24%) and maximum (64%) wombat's calories from fat corresponds to the seasonably varied sampling employed in the study.

To evaluate the effect of the elephants' disappearance from the Levant ca. 400 kyr ago, we chose two Levantine sites that exhibit a significant faunal record and reliable dating: GBY, dated to ca. 780 kyr, and Qesem Cave, dated to 400-200 kyr. Prior to an analysis, however, a short discussion of elephants is in order.

#### *Elephants*

Elephants (*Elephas antiquus*, or by another name Paleoloxodon antiquus, in the Pleistocene Levant) were by far the largest terrestrial animal, five times larger than the second largest animal found at GBY – the Hippopotamus. Elephants present a unique food package (see Table 3 above), the hunt of which is not particularly physically challenging and does not necessarily require mastery of sophisticated technology. It is an attractive hunting characteristic of elephants that they usually do not run away but rather depend on size, not speed, for their protection. Elephants are also relatively easy to locate in view of their repeated usage of known paths leading to water sources [91], [92] and thus easy to ambush or trap. It seems likely that spears were used to hunt elephants. Wooden spears have been found in Lower Paleolithic sites [93], [94] and are in common use by present day elephant hunters as established by Churchill [95] in a study of 96 HG groups. Several techniques are described for undisturbed human approach to elephants, indeed, often accompanied by the use of spears [95], [96]. One entails hiding in the bush next to an elephant path, surprising it from the rear and cutting its tendons. Another practice is that of trapping by digging a round pit of some 2 m in diameter and 3 m in depth in the midst of the elephant path, while spears are placed at the bottom and the top is covered with branches [96]. Another method entails scaring the elephant from behind against a barrier built of thick vines while forcing the animal to escape towards the barrier where it can be speared ([97]:115). Alternatively, a heavy object with pointed elements may be suspended over an elephant, such as a thick tree trunk, and dropped upon the animal as it passes below, thus breaking its spine ([97]:117). Elephants represent an ideal food package whose full utilization requires, according to anthropological reports (see, for example, [98] regarding the Mbuti), some form of sharing or long-term preservation by drying. Albeit the present lack of direct archaeological evidence, we believe that both practices were not beyond the capabilities of H. erectus. We have no knowledge of the size of the group that partook in the consumption of a single elephant during the Middle Pleistocene, but it seems plausible to consider the aggregation of several small groups in the case of a successful hunt. While we have no data regarding the extent to which a hunted elephant was utilized, in our view, the abundance of evidence for elephant utilization in Acheulian sites in itself is testimony that a significant part of the elephant's potential energetic value was extractable by H. erectus. This view is further supported not only by the fact that elephant bones bear cut marks but also by the fact that many bones were fractured to allow for marrow extraction, indicating further use of the naked bones for additional fat.

Elephant skeletal remains were found in many Acheulian (pre 400 kyr) sites of the Levant (see [28], [99] and references therein). The site of Holon yielded both Acheulian lithic assemblages and elephant remains. Originally assigned to a Middle Pleistocene Acheulian complex (ca. 500 kyr BP), the site was recently dated anew to ca. 200 kyr [100]. Commenting on this new suggestion, Bar-Yosef and Belmaker [101] say: “Unfortunately the [new – our addition] dates for Holon were retrieved from another geological exposure as the area of the original site lies below a major factory … and if the dates are accepted it would mean [that] … the Holon Acheulian was contemporary with the early Mousterian, which is untenable…”. We agree with Bar-Yosef and Belmaker that the 200 kyr date for Holon is unacceptable (and see [11], [101], [102]), which is in agreement with our notion that all Acheulian sites in the Levant are older than 400 kyr. On the other hand, there is not a single Acheulo-Yabrudian (400-200 kyr; [11], [101], [102]) site that yielded elephant remains apart from the uncontested case of the Acheulo-Yabrudian layer E at Tabun Cave with a single small tusk fragment ([103]:146). Remarkably, the disappearance of the elephant from the archeological record is also evident in Africa, both east and south, during the transition from the Acheulian to the Middle Stone Age ca. 250 kyr [29], [104].

While the disappearance of *Elephas antiquus* from the faunal record of the Levant by the end of the Acheulian prior to 400 kyr may have been the result of overkill, other causes cannot be ruled out (see [105]). Elephants can tolerate only up to 4% annual predation loss among adults due to their long gestation, infancy, and adolescence compared, for example, to suids that can tolerate up to 50% predation ([106]:138). This is especially significant considering the limited territorial Levantine buffer compared to Africa. Different researchers (e.g., [38]; and see [107]) have maintained a worldwide version of the anthropogenic excessive hunting hypothesis. Surovell et al. [108], for example, proposed such a continuous practice and anthropogenic worldwide Proboscidea extinction commencing in the Acheulian and ending in the New World, around 10 kyr BP. Lyons et al. [109], based on Coppens et al. [110], argued that in Africa, out of 12 species of elephant-like Proboscidea active during Early Pleistocene, only two survived to the Middle Pleistocene. Accepting a hypothesis of anthropogenic overkill, however, is not essential for the validity of our argument. For our purposes, it is only necessary to establish that elephants were an important dietary factor in the Acheulian and that they were absent in the Acheulo-Yabrudian.

Gesher Benot Yaaqov (GBY): GBY is a Lower-Middle Pleistocene Acheulian site in Israel, dated to 780 kyr BP. The site is located on the shore of an ancient paleo lake in the Upper Galilee [111]. The sedimentary sequence of GBY consists of some 34 m of lake-margin deposits, and the duration of the entire depositional sequence is estimated to be ca. 50 kyr [112]. Elephant remains are found in many occupation levels at the site ([26] and see our Table 4 below and Tables S3 and S4) and one particular context reflects evidence for a purposeful setting aimed at the extraction of the brain from an elephant skull [28]. The site is considered as an example of the migration of African H. erectus into the Levant and exhibits significant behavioral characteristics such as the spatial organization of space, the extensive use of basalt for handaxe production, nut-cracking stones, and more [113].

**Table 4:** The drastic dietary role of the elephant: GBY and Qesem Cave faunal caloric contribution and fat content compared.

Site and layer	Average calories per animal^1^	% fat of total faunal calories	% elephants in faunal assemblage	% elephants calories of total calories
**GBY** 2				
Layer V-6	166,688	46%	2%	59%
Layer V-5	191,659	46%	3%	60%
Layer JB	171,852	46%	2%	56%
Layer II-6	386,702	49%	6%	65%
**GBY, average**	**210,611**	**47%**	**3%**	**61%**
**Qesem Cave** 3				
Layer II	102,360	40%	None	None
Layer III	119,688	42%	None	None
Layer IV	102,861	40%	None	None
Layer V	82,080	38%	None	None
**Qesem Cave, average**	**99,996**	**40%**	None	None

Table Notes.

1 For missing information, data of representative animals of similar weight was considered ([87]: Appendix); see Table S3 and Table S4 for details.

2 Rabinovich and Biton ([26]:4, Table 2); data based on NISP (see Table S3 for details by size group).

3 Stiner et al. ([45]:Table 2). Data based on NISP.

Qesem Cave: Qesem Cave is a Middle Pleistocene site in central Israel, dated to 400-200 kyr BP. Cave inhabitants hunted cooperatively, bringing body-parts of fallow deer back to the cave, which were then butchered, shared, and – as evidenced by the use of fire throughout the cave's 7.5 m deep stratigraphy and the many burnt bones – eventually barbecued (see [9], [23], [45]). Plenteous cutting tools were produced at the site, most significantly flint blade knives made by an innovative and thoughtful technology [8], [114]. Moreover, our lithic analysis and the study of use-wear signs on flint artifacts indicate a set of cutlery manufactured to handle the different stages of butchering, defleshing, and meat cutting [44]. The preference for prime-age animals is apparent in this Acheulo-Yabrudian site, representing a unique human predator-prey relationship [115]. Notwithstanding, elephants are completely absent from Qesem Cave.

#### The faunas of the two sites compared

Despite representing two distinct cultural entities and although considerably distant in time, a comparison of faunal assemblages from GBY and Qesem Cave ([26]:4 and [45] respectively) reflects the heart of the matter discussed herein. While GBY includes a significant component of animals of the BSGA (e.g., elephants) as well as BSGB (Hippopotamus and Rhinoceros), the largest animal remains present at Qesem Cave are single bones (mostly teeth) of BSGB. Both sites include additional animals of body size groups BSGB – *Equus cab.*, *Bos prim*., BSGC – *Cervus ela.*, *Crevidae*, *Equus herm.*, *Sus scr.* and BSGD – *Dama cf. meso*.

Table 4 presents average faunal data of the two sites per layer based on NISP, which the researchers at both sites state best represents the animals' relative frequency [26], [45]. Note that layer II-6 at GBY is interpreted as a rapidly sealed context, most probably reflecting a well-preserved short-term occupation [116], while the other layers at GBY and the four samples of Qesem Cave represent distinct archaeological layers within the site's occupational history. The similarity of faunal patterns found at each of the two sites is telling while the difference between the sites remains highly significant across all archaeological layers. Fallow deer (Dama, BSGD), dominated both sites in terms of number of animals, but its contribution in calories is significantly different between the two sites. This difference is explained by the dominant calorie contribution of the elephants (both meat and fat) at GBY, amounting to over half of the calories in each of the GBY layers, and the average of 61% of consumed faunal calories.

## Results and Discussion

The following discussion deals with the disappearance of the elephant and the consequential bioenergetic significance of this occurrence as well as its human evolutionary implications.

### The disappearance of elephants from a bioenergetic viewpoint

Table 4 shows the crucial dietary role of the elephant, underlying the evolutionary model we suggest. It explains the nutritional dependence of both *H. erectus* and *H. sapiens* on large animals of the BSGA (elephant) and BSGB (*Equus cab.*, *Bos prim*.) classes. Maintaining the required level of fat consumption for *H. erectus* dictated the acquisition of animals with the average caloric fat content of 44% (Table 2). Table 3 shows that only large animals have the potential to make such a generous dietary contribution.

The disappearance of elephants from the diet of *H. erectus* in the Levant by the end of the Acheulian had two effects that interacted with each other, further aggravating the potential of *H. erectus* to contend with the new dietary requirements:

The absence of elephants, weighing five times the weight of Hippopotami and more than eighty times the weight of Fallow deer (Kob in Table 3), from the diet would have meant that hunters had to hunt a much higher number of smaller animals to obtain the same amount of calories previously gained by having elephants on the menu.

Additionally, hunters would have had to hunt what large (high fat content) animals that were still available, in order to maintain the obligatory fat percentage (44% in our model) since they would have lost the beneficial fat contribution of the relatively fat (49% fat) elephant. This ‘large animal’ constraint would have further increased the energetic cost of foraging.

Having to hunt a high number of faster and more evasive animals would have considerably increased DEE. Assuming a new and higher DEE and accepting that animal protein and vegetal calories were already at their upper limit, all additional calories would have had to be supplied by animal fat alone. In such a case utilizing, for example, Fallow deer, only 31% of the calories (Table 3 for Kob) of the animal would have been available, tripling the number of additional animals needed. DEE would have risen further to be supplied again only by the fat portion of the animal.

To illustrate the above points, the model we use provides a minimum for fat requirement originating in animal calories predicted as a function of the DEE. This relation is highly sensitive such that a small increase in DEE brings about a remarkable increase in the minimum of fat required from animal calories. Under our assumptions for *H. erectus*, the increase amounts to 2.7% per each 100 calories increased in DEE. This increase results in the need to obtain a greater number of animals whose fat content is relatively high. Even if we assume a higher maximal plant intake, for example 50% (compared to our original assumption of 38%), a 300 calorie increase in DEE demands that the initial 30% fat required from animal calories at the DEE of 2,704 calories rise to 43% at the DEE of 3,000 calories, further causing the need to search for a new, more energetically costly mix of prey selection.

The stress-causing cycle described above would have been significantly amplified by the pronounced effect of seasonality. Reduction of fat in animals during the dry season would have increased the stress imposed upon *H. erectus* when elephants began to disappear. A crucial advantage for a fat-dependent hominin in hunting large game is their higher retention of body fat throughout the year, even during dry seasons. Lindstedt [117] states that: “Large mammals have proportionately greater mass of body fat coupled with proportionately lower daily energy requirements” and goes on to mention elephants as an example for the relative advantage of large mammals that are able to retain body fat in areas where seasonality is pronounced regardless of temperatures. In this respect, the Levant is indeed characterized by a long dry season during which vegetation is minimal and animals are depleted of fat [4]. This, in terms of our model, represents the worst possible dietary scenario bearing the potential to accelerate the demise of *H. erectus*.

The *Homo* preference for large-game hunting has been a mainstay of our understanding of human prehistory [4], [48]. This preference, which we now suggest to view as a dependence by establishing an obligated fat dietary intake that is also very sensitive to increased DEE, must have emerged with *H. erectus* who had to exercise his “true role as a highly competitive member of the large carnivore community” ([106]:137).

Although the term “protein poisoning” is sometimes used to describe the physiological ceiling on protein consumption, we do not propose an acute physical deterioration of *H. erectus* in our model but rather an evolutionary process that occurred in response to the physiological stress that would have followed the disappearance of elephants and which would have gradually diminished fitness. Similar constraints may have prevailed among HG until quite recently. Cordain et al. ([53]:689) discuss the methods HG employ to circumvent the dietary protein ceiling, stating that “although an adoption of higher P-A (plant-animal) subsistence ratio by increasing plant food consumption appears to be the simplest solution … data from the *Ethnographic Atlas* clearly indicate that this approach was not the preferred solution … even where plant foods were available year round at lower latitudes”. They proceed to name hunting of larger prey as what “may have been the preferred solution”.

### Evolutionary Implications

An important outcome of the model is the fact that the obligatory fat requirement of *H. sapiens* (Table 2) was at least as restrictive as that of *H. erectus*. A lower body weight (smaller liver) decreased the amount of protein the body could digest and lower MQ resulted in a decrease in the amount of plant fiber one could digest. The observed reduction in body weight may have been consequential to the shift from the need to handle the heavy elephant to the need of obtaining and handling a greater number of lighter animals. The decreased body weight led to reduced DEE, which in turn prevented the obligatory dietary fat requirements from reaching impractical levels. *H. sapiens* was thus as dependent on large animals as *H. erectus*. From a locomotive point of view, the lighter body meant relatively (to weight) longer lower limbs [6], [118], which meant increased efficiency, allowing for a smaller increase in DEE despite the need to hunt a greater number of faster animals. Another aspect of locomotion may be of importance if one considers the endurance running (ER) hypothesis [119], [120] and assumes that modern humans adapted to the hunt of faster animals in warm climates by improving their ER capabilities (see also [121]).

The brain, conspicuously growing in size, allowed for higher cognitive and learning skills that may have lead to greater efficiency in hunting with the use of more sophisticated trucking and killing techniques as well as the employment of wider social skills [50], [76]. Potentially additional energy was also saved by adopting a home-based mobility strategy [122], [123].

Comparing the average calories per animal at GBY and Qesem Cave might lead to the conclusion that Qesem Cave dwellers had to hunt only twice as many animals than GBY dwellers. This, however, is misleading as obligatory fat consumption complicates the calculation of animals required. With the obligatory faunal fat requirement amounting to 49% of the calories expected to be supplied by the animal, Fallow deer with their caloric fat percentage of 31% (Kob in Table 3) would not have supplied enough fat to be consumed exclusively. Under dietary constraints and to lift their average fat consumption, the Qesem Cave dwellers would have been forced to hunt aurochs and horses whose caloric fat ratio amounts to 49% (the equivalent of buffalo in Table 3). The habitual use of fire at Qesem Cave, aimed at roasting meat [23], [45], may have reduced the amount of energy required for the digestion of protein, contributing to further reduction in DEE. The fact that the faunal assemblage at Qesem Cave shows significantly high proportions of burnt and fractured bones, typical of marrow extraction, is highly pertinent to the point. In addition, the over-representation of fallow deer skulls found at the site [9], [45] might imply a tendency to consume the brain of these prey animals at the cave. Combined, these data indicate a continuous fat-oriented use of prey at the site throughout the Acheulo-Yabrudian (400-200 kyr).

However, the average caloric fat percentage attributed to the animals at Qesem Cave – 40% – is still lower than the predicted obligatory fat requirements of faunal calories for *H. sapiens* in our model, amounting to 49% (Table 2). This discrepancy may have disappeared should we have considered in our calculations in Table 3 the previously mentioned preference for prime-age animals that is apparent at Qesem Cave [9], [45]. The analysis of Cordain's Caribou fat data ([124]: Figure 5) shows that as a strategy the selective hunting of prime-age bulls or females, depending on the season, could, theoretically, result in the increase of fat content as the percentage of liveweight by 76% from 6.4% to 11.3%, thus raising the caloric percentage of fat from animal sources at Qesem Cave. Citing ethnographic sources, Brink ([125]:42) writes about the American Indians hunters: “Not only did the hunters know the natural patterns the bison followed; they also learned how to spot fat animals in a herd. An experienced hunter would pick out the pronounced curves of the body and eye the sheen of the coat that indicated a fat animal”. While the choice of hunting a particular elephant would not necessarily be significant regarding the amount of fat obtained, this was clearly not the case with the smaller game. It is apparent that the selection of fat adults would have been a paying strategy that required high cognitive capabilities and learning skills.

We would like to return to the issue of the use of fire by *H. erectus*. While we accept the evidence attesting to the use of fire by *H. erectus* at the site of GBY [19], [20], [21], [126], and while we note the recently made suggestion by Beaumont [127] on the use of fire in South Africa some 1.7 my ago, we have no such evidence elsewhere. No other Lower Paleolithic site in the Levant, Africa, Europe, or Asia demonstrates the use of fire, while many Middle Paleolithic sites in these parts of the world show extensive use of fire. It thus appears to us that while fire was indeed practiced to some extent in specific and isolated Lower Paleolithic sites, it was not commonly and regularly used by *H. erectus* but only by his successors. We may, then, provisionally use the terminology suggested by Gowlett [25] and refer to Acheulian pre-400 kyr fire as ‘early fire’ and to the later, post-Acheulian in Levantine terms, as ‘late fire’ implying the continuous, habitual use of fire. We thus join other researchers [22], [23], [24], [25], [128] in suggesting that a wide-range habitual use of fire started around 400 kyr in both the Levant and Europe. The use of fire in the Acheulian, as far as the evidence goes, was only the case in particular sites and not as a major and common habit or cultural trait. This has changed around 400 kyr as evident at Qesem Cave and other contemporaneous sites. This is of significance here since it is generally agreed that cooking had significant evolutionary survival advantages and greatly changed the feeding behaviors of humans [129]. Thermal processing of meat has advantages in increasing meat intake and amplifying the energy available from meat; rendering proteins more digestible through denaturation (see [130], [131]); lowering the cost of digestion through food softening and reducing immune regulation by eliminating food borne pathogens; positively altering the fat's energy value due to easy digestion and faster absorption; possibly allowing long-term meat preservation; and more. While the effect of cooking on plant foods and the energy gained from cooked plants as presented by Carmody and Wrangham [129] is significant, it is not enough to significantly lower fat dependability. The ample isotopic, genetic, and ethnographic evidence presented above casts doubts on a significant consumption of plant foods in Paleolithic times beyond the 32% caloric contribution determined by our model.

In summary, the notion that shifts in human life histories, accompanied by improved intelligence, are an evolutionary response to a dietary shift towards high-quality food resources that are difficult to acquire has already been suggested by Kaplan [7]. Our model is innovative in that it suggests a mechanism for such a dietary shift that could have propelled hominins to a new evolutionary stage.

### Conclusion

For more than two decades a view dominated anthropological discussions that all modern human variation derived from Africa within a relatively recent chronological framework. Recent years challenged this paradigm with new discoveries from Europe, China, and other localities, as well as by new advances in theory and methodology. These developments are now setting the stage for a new understanding of the human story in general and the emergence of modern humans in particular (e.g., [1], [39], [132], [133], [134], [135], [136], [137], [138], [139], [140], [141], [142], [143], [144], [145], [146]). In this respect, the Qesem hominins may play an important role. Analysis of their dental remains [1] suggests a much deeper time frame between at least some of the ancestral populations and modern humans than that which is assumed by the “Out of Africa” model. This, combined with previous genetic studies (e.g., [147], [148], [149], [150]), lends support to the notion of assimilation (e.g., [144]) between populations migrating “out of Africa” and populations already established in these parts of Eurasia.

It is still premature to indicate whether the Qesem hominin ancestors evolved in Africa prior to 400 kyr [136], developed blade technologies [151], [152], and then migrated to the Levant to establish the new and unique Acheulo-Yabrudian cultural complex; or whether (as may be derived from our model) we face a local, Levantine emergence of a new hominin lineage. The plethora of hominins in the Levantine Middle Paleolithic fossil record (Qafzeh, Skhul, Zuttiyeh, Tabun) and the fact that the Acheulo-Yabrudian cultural complex has no counterparts in Africa may hint in favor of local cultural and biological developments. This notion gains indirect support from the Denisova finds that raise the possibility that several different hominin groups spread out across Europe and Asia for hundreds of thousands of years, probably contributing to the emergence of modern human populations [153], [154], [155].

It should not come as a surprise that *H. erectus*, and its successors managed, and in fact evolved, to obtain a substantial amount of the densest form of nutritional energy available in nature – fat – to the point that it became an obligatory food source. Animal fat was an essential food source necessary in order to meet the daily energy expenditure of these Pleistocene hominins, especially taking into account their large energy-demanding brains. It should also not come as a surprise that for a predator, the disappearance of a major prey animal may be a significant reason for evolutionary change. The elephant was a uniquely large and fat-rich food-package and therefore a most attractive target during the Levantine Lower Palaeolithic Acheulian. Our calculations show that the elephant's disappearance from the Levant just before 400 kyr was significant enough an event to have triggered the evolution of a species that was more adept, both physically and mentally, to obtain dense energy (such as fat) from a higher number of smaller, more evasive animals. The concomitant emergence of a new and innovative cultural complex – the Acheulo-Yabrudian, heralds a new set of behavioral habits including changes in hunting and sharing practices [9], [23], [45] that are relevant to our model.

Thus, the particular dietary developments and cultural innovations joined together at the end of the Lower Paleolithic period in the Levant, reflecting a link between human biological and cultural/behavioral evolution. If indeed, as we tried to show, the dependence of humans on fat was so fundamental to their existence, the application is made possible, perhaps after some refinement, of this proposed bioenergetic model to the understanding of other important developments in human evolutionary history.

## Supporting Information

Table S1 **Calculation of Daily Energy Expenditure (DEE).**
(DOC)

Table S2 **Protein and fat content of African Game Animals.**
(DOC)

Table S3 **Comparison of relative contribution of fat and protein between two Levantine sites - GBY and Qesem Cave.**
(XLS)

Table S4 **Number of NISP per layer.**
(DOC)

## References

[1] Hershkovitz I, Smith P, Sarig R, Quam R, Rodríguez L (2011). Middle Pleistocene dental remains from Qesem Cave, Israel.. American Journal of Physical Anthropology.

[2] Aiello LC, Wheeler P (1995). The expensive-tissue hypothesis: The brain and the digestive system in human and primate evolution.. Current Anthropology.

[3] Bunn HT, Ezzo JA (1993). Hunting and scavenging by Plio-Pleistocene hominids: Nutritional constraints, archaeological patterns, and behavioral implications.. J Archaeol Sci.

[4] Speth JD (2010). The paleoanthropology and archaeology of big-game hunting protein, fat, or politics?.

[5] McHenry HM, Ruse M, Travis J (2009). Human evolution.. Evolution: The first four billion years.

[6] Steudel-Numbers KL, Weaver TD, Wall-Scheffler CM (2007). The evolution of human running: effects of changes in lower-limb length on locomotor economy.. Journal of Human Evolution.

[7] Kaplan H, Gangestad S, Gurven M, Lancaster J, Mueller T, Roebroeks W (2007). The evolution of diet, brain and life history among primates and humans.. Guts and brains an integrative approach to the hominin record.

[8] Barkai R, Lemorini C, Shimelmitz R, Lev Z, Stiner M (2009). A blade for all seasons? Making and using Amudian blades at Qesem Cave, Israel.. Human Evolution.

[9] Stiner M, Gopher A, Barkai R (2009). Cooperative hunting and meat sharing 400-200 kya at Qesem Cave, Israel.. Proc Natl Acad Sci U S A.

[10] Barkai R, Gopher A, Lauritzen SE, Frumkin A (2003). Uranium series dates from Qesem Cave, Israel, and the end of the Lower Palaeolithic.. Nature.

[11] Gopher A, Ayalon A, Bar-Matthews M, Barkai R, Frumkin A (2010). The chronology of the Late Lower Paleolithic in the Levant: U series dates of speleothems from Middle Pleistocene Qesem cave, Israel.. Quaternary Geochronology.

[12] Aiello LC, Wells JCK (2002). Energetics and the evolution of the genus Homo.. Annual Review of Anthropology.

[13] Antón SC (2003). Natural history of Homo erectus.. Yearbook of Physical Anthropology.

[14] Gilberst WH, Asfaw B (2008). Homo erectus. Pleistocene evidence from the Middle Awash.

[15] Bello SM, Parfitt SA, Stringer C (2009). Quantitative micromorphological analyses of cut marks produced by ancient and modern handaxes.. J Archaeol Sci.

[16] Machin AJ, Hosfield RT, Mithen SJ (2007). Why are some handaxes symmetrical? Testing the influence of handaxe morphology on butchery effectiveness.. J Archaeol Sci.

[17] Wrangham R, Carmody RN (2010). Human adaptation to the control of fire.. Evolutionary Anthropology: Issues, News and Reviews.

[18] Wrangham RW, Jones JH, Laden G, Pilbeam D, Conklin-Brittain N (1999). The raw and the stolen: cooking and the ecology of human origins.. Current Anthropology.

[19] Alperson-Afil N (2008). Continual fire-making by hominins at Gesher Benot Ya'akov, Israel.. Quaternary Science Reviews.

[20] Alperson-Afil N, Goren-Inbar N (2010). The Acheulian site of Gesher Benot Ya'aqov volume II: Ancient flames and controlled use of fire.

[21] Goren-Inbar N, Alperson N, Kislev ME, Simchoni O, Melamed Y (2004). Evidence of hominin control of fire at Gesher Benot Ya'aqov, Israel.. Science.

[22] Gamble C, Gowlett JAJ, Dunbar R (2011). The social brain and the shape of the Palaeolithic.. Cambridge Archaeological Journal.

[23] Karkanas P, Shahack-Gross R, Ayalon A, Bar-Matthews M, Barkai R (2007). Evidence for habitual use of fire at the end of the Lower Paleolithic: Site formation processes at Qesem Cave, Israel.. Journal of Human Evolution.

[24] Roebroeks W, Villa P (2011). On the earliest evidence for habitual use of fire in Europe.. Proc Natl Acad Sci U S A.

[25] Gowlett JAJ, Dunbar R, Gamble C, Gowlett JAJ (2010). Firing up the social brain, in. Social brain, distributed mind (Proceedings of the British Academy).

[26] Rabinovich R, Biton R (2011). The Early-Middle Pleistocene faunal assemblages of Gesher Benot Ya'aqov: Inter-site variability.. Journal of Human Evolution.

[27] Delagnes A, Lenoble A, Harmand S, Brugal J, Prat S (2006). Interpreting pachyderm single carcass sites in the African Lower and Early Middle Pleistocene record: A multidisciplinary approach to the site of Nadung'a 4 (Kenya).. Journal of Anthropological Archaeology.

[28] Goren-Inbar N, Lister A, Werker E, Chech M (1994). A butchered elephant skull and associated artifacts from the Acheulian site of Gesher Benot Ya'aqov, Israel.. Paleorient.

[29] Klein RG (1988). The archaeological significance of animal bones from Acheulean sites in southern Africa.. The African Archaeological Review.

[30] Yravedra J, Domínguez-Rodrigo M, Santonja M, Pérez-Gonzálezc A, Panera J (2010). Cut marks on the Middle Pleistocene elephant carcass of Aridos 2 (Madrid, Spain).. J Archaeol Sci.

[31] Gaudzinski S, Turner E, Anzidei AP, Alvarez-Fernández E, Arroyo-Cabrales J (2005). The use of proboscidean remains in every-day Palaeolithic life.. Quaternary International.

[32] Clark JD, Beyene Y, WoldeGabriel G, Hart WK, Renne PR (2003). Stratigraphic, chronological and behavioural contexts of a Pleistocene Homo sapiens from Middle Awash, Ethiopia.. Nature.

[33] McDougall I, Brown FH, Fleagle JG (2005). Stratigraphic placement and age of modern humans from Kibish, Ethiopia.. Nature.

[34] White TD, Asfaw B, DeGusta D, Gilbert H, Richards GD (2003). Pleistocene Homo sapiens from Middle Awash, Ethiopia.. Nature.

[35] Beyene Y (2010). Herto brains and minds: Behaviour of early Homo sapiens from the Middle Awash.. Proceedings of the British Academy.

[36] Mcbrearty S (2003). Patterns of technological change at the origin of Homo sapiens.. Journal of Human Evolution.

[37] Klein RG, Avery G, Cruz-Uribe K, Steele TE (2007). The mammalian fauna associated with an archaic hominin skullcap and later Acheulean artifacts at Elandsfontein, Western Cape Province, South Africa.. Journal of Human Evolution.

[38] Martin PS, Martin PS, Klein RG (1984). Overkill: The global model.. Quaternary extinctions: A prehistoric revolution.

[39] Schwartz JH, Tattersal I (2010). Fossil evidence for the origins of Homo sapiens.. Yearbook of Physical Anthropology.

[40] Mounier A, Marchal F, Condemi S (2009). Is Homo heidelbergensis a distinct species? New insight on the Mauer mandible.. Journal of Human Evolution.

[41] Hublin JJ (2009). The origins of Neandertals.. Proc Natl Acad Sci U S A.

[42] White M, Pettitt P (2011). The British Middle Palaeolithic: An interpretative synthesis of Neanderthal occupation at the Northwestern edge of the Pleistocene world.. Journal of World Prehistory.

[43] Mellars P, French J (2011). Tenfold population increase in Western Europe at the Neandertal-to-modern human transition.. Science.

[44] Barkai R, Lemorini C, Gopher A (2010). Palaeolithic cutlery 400 000-200 000 years ago: Tiny meat-cutting tools from Qesem Cave, Israel. Antiquity 84(325).. http://antiquity.ac.uk/projgall/barkai325/.

[45] Stiner MC, Gopher A, Barkai R (2011). Hearth-side socioeconomics, hunting and paleoecology during the late Lower Paleolithic at Qesem Cave, Israel.. Journal of Human Evolution.

[46] Foley RA, Lee PC (1991). Ecology and energetics of encephalization in hominid evolution.. Philosophical Transactions of the Royal Society of London B.

[47] Cunnane SC, Crawford MA (2003). Survival of the fattest: fat babies were the key to evolution of the large human brain.. Comparative Biochemistry and Physiology Part A.

[48] Smil V (2002). Eating meat: Evolution, patterns, and consequences.. Population and Development Review.

[49] Stanford C, Bunn H (2001). Meat eating and human evolution.

[50] Stanford CB (1999). The hunting apes: Meat eating and the origins of human behavior.

[51] Noli D, Avery G (1988). Protein poisoning and coastal subsistence.. J Archaeol Sci.

[52] Speth JD, Spielmann KA (1983). Energy source, protein metabolism , and subsistence strategies.. Journal of Anthropological Archaeology.

[53] Cordain L, Miller JB, Eaton SB, Mann N, Holt SH (2000). Plant-animal subsistence ratios and macronutrient energy estimations in worldwide hunter-gatherer diets.. The American Journal of Clinical Nutrition.

[54] Speth JD (1989). Early hominid hunting and scavenging: the role of meat as an energy source.. Journal of Human Evolution.

[55] Eades MR, Eades MD (1996). Protein power.

[56] NutritionData website.. http://www.nutritiondata.com.

[57] Metges CC, Barth CA (2000). Metabolic consequences of a high dietary-protein intake in adulthood: assessment of the available evidence.. The Journal of Nutrition.

[58] Rudman D, DiFulco TJ, Galambos JT, Smith RB, Salam AA (1973). Maximal rates of excretion and synthesis of urea in normal and cirrhotic subjects.. Journal of Clinical Investigation.

[59] Milton K, Harris M, Ross EB (1987). Primate diets and gut morphology: Implications for hominid evolution.. Food and evolution: Toward a theory of human food habits.

[60] Bock J (2002). Learning, life history, and productivity: Children?s lives in the Okavango Delta, Botswana.. Human Nature.

[61] De Caluwé E, Halamová K, Van Damme P (2010). Adansonia digitata L. A review of traditional uses, phytochemistry and pharmacology.. Afrika Focus.

[62] Stiner MC, Kuhn SL, Hublin JJ, Richards MP (2009). Paleolithic diet and the division of labor in Mediterranean Eurasia.. The evolution of hominin diets integrating approaches to the study of Palaeolithic subsistence.

[63] Coursey DG (1973). Hominid evolution and hypogeous plant foods.. Man.

[64] Hatley T, Kappleman J (1980). Bears, pigs, and Pleistocene hominids: a case for the exploitation of belowground resources.. Human Ecology.

[65] Conklin-Brittain NL, Wrangham R, Smith CC, Ungar PS, Teaford MF (2002). A two-stage model of increased dietary quality in early hominid evolution: The role of fiber.. Human diet: Its origin and evolution.

[66] Ungar PS, Grine FE, Teaford MF (2006). Diet in early Homo: a review of the evidence and a new model of adaptive versatility.. Annual Review of Anthropology.

[67] McHenry HM, Grine FE (1988). New estimates of body weight in early hominids and their significance to encephalization and megadontia in robust australopithecines.. The evolutionary history of the “robust” australopithecines.

[68] Ungar P (1998). Dental allometry, morphology, and wear as evidence for diet in fossil primates.. Evolutionary Anthropology.

[69] Ungar P (2004). Dental topography and diets of Australopithecus Afarensis and early Homo.. Journal of Human Evolution.

[70] Roebroeks W, Villa P (2011). Reply to Sandgathe et al.: Neandertal use of fire.. Proc Natl Acad Sci U S A.

[71] Sandgathe DM, Dibble HL, Goldberg P, McPherron SP, Turq A (2011). Timing of the appearance of habitual fire use.. Proc Natl Acad Sci U S A.

[72] Lee-Thorp JA, Sponheimer MB (2006). Contributions of biogeochemistry to understanding hominin dietary ecology.. Yearbook of Physical Anthropology.

[73] Richards MP, Trinkaus E (2009). Isotopic evidence for the diets of European Neanderthals and early modern humans.. Proc Natl Acad Sci U S A.

[74] Richards MP, Hublin JJ, Richards MP (2009). Stable isotope evidence for European Upper Paleolithic human diets.. The evolution of hominin diets integrating approaches to the study of Palaeolithic subsistence.

[75] Ströhle A, Hahn A (2011). Diets of modern hunter-gatherers vary substantially in their carbohydrate content depending on ecoenvironments: results from an ethnographic analysis.. Nutrition.

[76] Kaplan H, Hill K, Lancaster J, Hurtado AM (2000). A theory of human life history evolution: Diet, intelligence, and longevity.. Evolutionary Anthropology.

[77] Hancock AM, Witonsky DB, Ehler E, Alkorta-Aranburu G, Beall C (2010). Colloquium paper: Human adaptations to diet, subsistence, and ecoregion are due to subtle shifts in allele frequency.. Proc Natl Acad Sci U S A.

[78] Perry GH, Dominy NJ, Claw KG, Lee AS, Fiegler H (2007). Diet and the evolution of human amylase gene copy number variation.. Nature Genetics.

[79] Lucas PW, Sui Z, Ang KY, Tan HTW, King SH, Hublin JJ, Richards MP (2009). Meals versus snacks and the human dentition and diet during the Paleolithic.. The evolution of hominin diets integrating approaches to the study of Palaeolithic subsistence.

[80] Ungar PS, Krueger KL, Blumenschine RJ, Njau J, Scott RS (2011). Dental microwear texture analysis of hominins recovered by the Olduvai Landscape Paleoanthropology Project, 1995–2007.. Journal of Human Evolution.

[81] Ho KJ, Biss K, Mikkelson B, Lewis LA, Taylor CB (1971). The Masai of East Africa: Some unique biological characteristics.. Archives of Pathology.

[82] McClellan WS, Du Bois EF (1930). Clinical calorimetry, XLV, Prolonged meat diets with a study of kidney function and ketosis.. Journal of Biological Chemistry.

[83] Halton TL, Hu FB (2004). The effects of high protein diets on thermogenesis, satiety and weight loss: A critical review.. Journal of the American College of Nutrition.

[84] Birkett A, Muir J, Phillips J, Jones G, O'Dea K (1996). Resistant starch lowers fecal concentrations of ammonia and phenols in humans.. The American Journal of Clinical Nutrition.

[85] Jorgensen H, Gabert VM, Hedemann MS, Jensen SK (2000). Digestion of fat does not differ in growing pigs fed diet containing fish oil, rapeseed oil, or coconut oil.. The Journal of Nutrition.

[86] Popovich DG, Jenkins DJ, Kendall CW, Dierenfeld ES, Carroll RW (1997). The western lowland gorilla diet has implications for the health of humans and other hominoids.. The Journal of Nutrition.

[87] Ledger HP (1968). Body composition as a basis for a comparative study of some East African mammals. Comparative nutrition of wild animals.. Proceedings of a symposium held at the zoological society of London on 10 and 11 November 1966.

[88] Cordain L, Watkins BA, Mann NJ (2001). Fatty acid composition and energy density of foods available to African hominids. Evolutionary implications for human brain development.. World Rev Nutr Diet.

[89] Crawford MA, Gale MM, Woodford MH, Casped NM (1970). Comparative studies on fatty acid composition of wild and domestic meats.. Int J Biochem.

[90] Woolnough AP, Foley WJ, Johnson CN, Evans M (1997). Evaluation of techniques for indirect measurement of body composition in a free-ranging large herbivore, the Southern Hairy-nosed Wombat.. Wildlife Research.

[91] Loarie SR, Aarde RJV, Pimm SL (2009). Fences and artificial water affect African savannah elephant movement patterns.. Biological Conservation.

[92] Shannon G, Matthews WS, Page BR, Parker GE, Smith RJ (2009). The affects of artificial water availability on large herbivore ranging patterns in savanna habitats: a new approach based on modelling elephant path distributions.. Diversity and Distributions.

[93] Movius HL (1950). A wooden spear of third interglacial age from Lower Saxony source.. Southwestern Journal of Anthropology.

[94] Thieme H (1997). Lower Palaeolithic hunting spears from Germany.. Nature.

[95] Churchill SE (1993). Weapon technology, prey size selection, and hunting methods in modern hunter-gatherers: Implications for hunting in the Palaeolithic and Mesolithic.. Archeological Papers of the American Anthropological Association.

[96] Marks S (1976). Large mammals and a brave people.

[97] Du Chaillu B (1868).

[98] Ichikawa M (1983). An examination of the hunting-dependent life of The Mbuti pygmies, Eastern Zaire.. Human Evolution.

[99] Marder O, Malinsky-Buller A, Shahack-Gross R, Ackermann O, Ayalon A (2011). Archaeological horizons and fluvial processes at the Lower Paleolithic open-air site of Revadim (Israel).. Journal of Human Evolution.

[100] Porat N, Chazan M, Kolska-Horwitz L (2007). Luminescence and electron spin resonance dating.. Holon, a Lower Paleolithic Site in Israel (American School of Prehistoric Research Bulletin).

[101] Bar-Yosef O, Belmaker M (2011). Early and Middle Pleistocene Faunal and hominins dispersals through Southwestern Asia.. Quaternary Science Reviews.

[102] Bar-Yosef O, Garfinkel Y (2008). The Prehistory of Israel.

[103] Bate DMA, Garrod DAE, Bate DMA (1937). Paleontology: The fossil fauna of Wady el-Mughara caves.. The Stone Age of Mount Carmel, Part 2.

[104] Klein RG (1983). The Stone Age prehistory of Southern Africa.. Annual Review of Anthropology.

[105] Koch PL, Barnosky AD (2006). Late quaternary extinctions: State of the debate.. Annual Review of Ecology Evolution and Systematics.

[106] Guthrie R, Roebroeks W (2007). Haak en Steek – The tool that allowed hominins to colonize the African savannah and flourish there.. Guts and brains an integrative approach to the hominin record.

[107] Alroy J (2001). A multispecies overkill simulation of the end-Pleistocene megafauna.. Science.

[108] Surovell T, Waguespack N, Brantingham PJ (2005). Global archaeological evidence for proboscidean overkill.. Proc Natl Acad Sci U S A.

[109] Lyons SK, Smith FA, Brown JH (2004). Of mice, mastodons and men: human-mediated extinctions on four continents.. Evolutionary Ecology Research.

[110] Coppens Y, Maglio VJ, Madden CT, Beden M, Maglio VJ, Cooke HBS (1978). Proboscidea.. Evolution of African mammals.

[111] Goren-Inbar N, Feibel CS, Verosub KL, Melamed Y, Kislev ME (2000). Pleistocene milestones on the out-of-Africa corridor at Gesher Benot Ya'aqov, Israel.. Science.

[112] Sharon G, Alperson-Afil N, Goren-Inbar N (2011). Cultural conservatism and variability in the Acheulian sequence of Gesher Benot Ya'aqov.. Journal of Human Evolution.

[113] Goren-Ibnar N (2011). Culture and cognition in the Acheulian industry: A case study from Gesher Benot Yaaqov.. Philosophical Transactions of the Royal Society of London B.

[114] Shimelmitz R, Gopher A, Barkai R (2011). Systematic blade production at Late Lower Paleolithic (400-200 kyr) Qesem Cave, Israel.. Journal of Human Evolution.

[115] Stiner MC (1990). The use of mortality patterns in archaeological studies of hominid predatory adaptations.. Journal of Anthropological Archaeology.

[116] Alperson-Afil N, Sharon G, Kislev M, Melamed Y, Zohar I (2009). Spatial organization of hominin activities at Gesher Benot Ya'aqov, Israel.. Science.

[117] Lindstedt SL, Boyce MS (1985). Seasonality, fasting endurance, and body size in mammals.. The American Naturalist.

[118] McHenry HM (1992). Body size and proportions in early hominids.. American Journal of Physical Anthropology.

[119] Lieberman DE, Bramble DM, Raichlen DA, Shea JJ, Grine FE, Fleagle JG, Leakey RE (2009). Brains, brawn, and the evolution of human endurance running capabilities.. The first humans: Origins and early evolution of the genus Homo.

[120] Ruxton GD, Wilkinson DM (2011). Thermoregulation and endurance running in extinct hominins: Wheeler's models revisited.. Journal of Human Evolution.

[121] Graves RR, Lupo AC, McCarthy RC, Wescott DJ, Cunningham DL (2010). Just how strapping was KNM-WT 15000?. Journal of Human Evolution.

[122] Marin A (2011). Palaeolithic human subsistence in Mount Carmel (Israel). A taphonomic assessment of Middle and Early Upper Palaeolithic faunal remains from Tabun, Skhul and el-Wad.. International Journal of Osteoarchaeology.

[123] Rolland N (2004). Was the emergence of home bases and domestic fire a punctuated event? A review of the Middle Pleistocene record in Eurasia.. Asian perspectives.

[124] Cordain L, Eaton SB, Sebastian A, Mann N, Lindeberg S (2005). Origins and evolution of the Western diet: health implications for the 21st century.. The American Journal of Clinical Nutrition.

[125] Brink J (2008). Imagining head-smashed-in Aboriginal buffalo hunting on the northern plains.

[126] Richter DN, Alperson-Afil N, Goren-Inbar N (2011). Employing TL methods for the verification of macroscopically determined heat alteration of flint artefacts from Palaeolithic contexts.. Archaeometry.

[127] Beaumont PB (2011). The edge: More on fire-making by about 1.7 million years ago at Wonderwerk Cave in South Africa.. Current Anthropology.

[128] Preece RC, Gowlett JAJ, Parfitt SA, Bridgland DR, Lewis SG (2006). Humans in the Hoxnian: habitat, context and fire use at Beeches Pit, West Stow, Suffolk, UK.. Journal of Quaternary Science.

[129] Carmody RN, Wrangham RW (2009). The energetic significance of cooking.. Journal of Human Evolution.

[130] Gaman PM, Sherrington KB (1996). The science of food: An introduction to food science, nutrition and microbiology.

[131] Lawrie RA (1991). Meat science.

[132] Carbonell E, Bermúdez de Castro JM, Parés JM, Pérez-González A, Cuenca-Bescós G (2008). The first hominin of Europe.. Nature.

[133] Cyran K, Kimmel M (2010). Alternatives to the Wright-Fisher model: The robustness of mitochondrial Eve dating.. Theoretical Population Biology.

[134] Dennel R (2010). Early Homo sapiens in China.. Nature.

[135] Dennell RW, Martinón-Torres M, Bermúdez de Castro JM (2009). Out of Asia: The initial colonisation of Europe in the Early and Middle Pleistocene.. Quaternary International.

[136] Endicott P, Ho S, Stringer C (2010). Using genetic evidence to evaluate four palaeoanthropological hypotheses for the timing of Neanderthal and modern human origins.. Journal of Human Evolution.

[137] Hou YM, Zhao LX (2010). An archaeological view for the presence of early humans in China.. Quaternary International.

[138] Klein RG (2008). Out of Africa and the evolution of human behavior.. Evolutionary Anthropology.

[139] Liu W, Jin CZ, Zhang YQ, Cai YJ, Xing S (2010). Human remains from Zhirendong, South China, and modern human emergence in East Asia.. Proc Natl Acad Sci U S A.

[140] Rightmire GP (2008). Homo in the Middle Pleistocene: Hypodigms, variation and species recognition.. Evolutionary Anthropology.

[141] Wood B (2010). Reconstructing human evolution: Achievements, challenges, and opportunities.. Proc Natl Acad Sci U S A.

[142] Wood B, Lonergan N (2008). The hominin fossil record: taxa, grades and clades.. Journal of Anatomy.

[143] Hawks JD, Wolpoff MH (2001). The four faces of Eve: hypothesis compatibility and human origins.. Quaternary International.

[144] Smith FH, Jankovic I, Karavanic I (2005). The assimilation model, modern human origins in Europe, and the extinction of Neandertals.. Quaternary International.

[145] Martinón-Torres M, Dennell R, Bermúdez de Castro JM (2011). The Denisova hominin need not be an out of Africa story.. Journal of Human Evolution.

[146] Cartmill M, Smith FH (2009).

[147] Harding R, Donnelly P, Tavare S (1997). Lines of descent from mitochondrial eve: An evolutionary look at coalescence.. Progress in population genetics and human evolution.

[148] Harding R, Fullerton S, Griffiths R, Bond J, Cox M (1997). Archaic African and Asian lineages in the genetic ancestry of modern humans.. American Journal of Human Genetics.

[149] Templeton A (2002). Out of Africa again and again.. Nature.

[150] Yu N, Zhao Z, Fu Y, Sambuughin N, Ramsay M (2001). Global patterns of human DNA sequence variation in a 10-kb region on Chromosome 1.. Molecular Biology and Evolution.

[151] Johnson CR, Mcbrearty S (2010). 500,000 year old blades from the Kapthurin Formation, Kenya.. Journal of Human Evolution.

[152] Porat N, Chazan M, Grün R, Aubert M, Eisenmann V (2010). New radiometric ages for the Fauresmith industry from Kathu Pan, southern Africa: Implications for the Earlier to Middle Stone Age transition.. J Archaeol Sci.

[153] Krause J, Fu Q, Good JM, Viola B, Shunkov MV (2010). The complete mitochondrial DNA genome of an unknown hominin from southern Siberia.. Nature.

[154] Reich D, Green RE, Kircher M, Krause J, Patterson N (2010). Genetic history of an archaic hominin group from Denisova Cave in Siberia.. Nature.

[155] Reich D, Patterson N, Kircher M, Delfin F, Nandineni M (2011). Admixture and the first modern human dispersals into Southeast Asia and Oceania.. Am J Hum Genet.

[156] Leonard WR, Robertson ML (1997). Comparative primate energetics and hominid evolution.. American Journal of Physical Anthropology.

[157] Byers D, Ugan A (2005). Should we expect large game specialization in the late Pleistocene? An optimal foraging perspective on early Paleoindian prey choice.. J Archaeol Sci.

[158] Christiansen P (2004). Body size in proboscideans, with notes on elephant metabolism.. Zoological Journal of the Linnean Society.

[159] Pitts GC, Bullard TR (1968). Some interspecific aspects of body composition in mammals. Body composition in animals and man.

