# Supplementary material for: Man the Fat Hunter: The Demise of Homo erectus and the Emergence of a New Hominin Lineage in the Middle Pleistocene (ca. 400 kyr) Levant
Source: PLoS One. 2011 Dec 9;6(12):e28689. doi: 10.1371/journal.pone.0028689 (PMC3235142; doi:10.1371/journal.pone.0028689)
Supplement: Table S1 — Calculation of Daily Energy Expenditure (DEE). (DOC) [file pone.0028689.s001.doc]

##### Table S1. Calculation of Daily Energy Expenditure (DEE)^1^

| Average m+f | Weight (kg)^2^ | Klieber RMR^3^ | TEE (DEE)^4^ |
| --- | --- | --- | --- |
| *H. erectus* | 61 | 1528 | 2704 |
| *H. sapiens* | 53.5 | 1385 | 2451 |

Table Notes

1. Also known as Total Energy Expenditure (TEE). Data in this table are based on TEE/BMR (Total Energy Expenditure/Basic Metabolic Rate) ratio for modern HG groups as per [1]: 275, Table 6.
2. Average weights for male and female per McHenry 2009:258
3. Resting Metabolic Rate (RMR) = Weight^0.75X70
4. TEE = RMR x 1.77

# References for Supporting Table 1

1. Leonard WR, Robertson ML (1997) Comparative primate energetics and hominid evolution. American Journal of Physical Anthropology 102(2): 265-281.
