# Supplementary material for: Man the Fat Hunter: The Demise of Homo erectus and the Emergence of a New Hominin Lineage in the Middle Pleistocene (ca. 400 kyr) Levant
Source: PLoS One. 2011 Dec 9;6(12):e28689. doi: 10.1371/journal.pone.0028689 (PMC3235142; doi:10.1371/journal.pone.0028689)
Supplement: Table S2 — Protein and fat content of African Game Animals. (DOC) [file pone.0028689.s002.doc]

##### Table S2. Protein and fat content of African Game Animals^1^

| Animal | M/F | Liveweight | Carcass fat | Offal fat | Marrow fat | Total fat | Total protein | Fat calories^2^ | Protein calories | % of fat from liveweight | % of protein from liveweight | % fat calories | Total calories | Weight ratio of elephant to animal | Calorie ratio of elephant to animal |
| --- | --- | --- | --- | --- | --- | --- | --- | --- | --- | --- | --- | --- | --- | --- | --- |
| Elephant (*Elephas antiquus*) |  | 6952 |  |  |  | 286.9 | 545.6 | 2,116,959 | 2,182,540 | 4.13% | 7.85% | 49% | 4,299,499 |  |  |
| Hipopotamus Amp | M+F | 1383 | 51.55 | 16.2 | 1.7 | 69.45 | 100.3 | 512,541 | 401,200 | 5.02% | 7.25% | 56% | 913,741 | 5 | 5 |
| Buffalo | M | 753 | 21.3 | 9 | 0.77 | 31.07 | 59.1 | 229,297 | 236,400 | 4.13% | 7.85% | 49% | 465,697 | 9 | 9 |
| Eland | M | 508 | 12.7 | 9.2 | 0.56 | 22.46 | 49.8 | 165,755 | 199,200 | 4.42% | 9.80% | 45% | 364,955 | 14 | 12 |
| Wildebeest | M+F | 217.5 | 8.35 | 3.9 | 0.31 | 12.56 | 22.2 | 92,693 | 88,800 | 5.77% | 10.21% | 51% | 181,493 | 32 | 24 |
| Oryx | M+F | 168.5 | 4.85 | 1.65 | 0.16 | 6.66 | 17 | 49,151 | 68,000 | 3.95% | 10.09% | 42% | 117,151 | 41 | 37 |
| Topi | M+F | 117.5 | 1.35 | 0.8 | 0.05 | 2.2 | 12 | 16,236 | 48,000 | 1.87% | 10.21% | 25% | 64,236 | 59 | 67 |
| Kob | M+F | 79.4 | 1.5 | 0.75 | 0.06 | 2.31 | 9.6 | 17,048 | 38,400 | 2.91% | 12.09% | 31% | 55,448 | 88 | 78 |
| Lesser Kudu | M | 92 | 1.9 | 0.9 | 0.07 | 2.87 | 9.6 | 21,181 | 38,400 | 3.12% | 10.43% | 36% | 59,581 | 76 | 72 |
| Warthog | M+F | 88 | 0.8 | 0.45 | 0.04 | 1.29 | 8.4 | 9,520 | 33,600 | 1.47% | 9.55% | 22% | 43,120 | 79 | 100 |
| Grant's Gazelle | M+F | 50.7 | 1.1 | 0.55 | 0.04 | 1.69 | 6.1 | 12,472 | 24,400 | 3.33% | 12.03% | 34% | 36,872 | 137 | 117 |
| Impala | M+F | 49.4 | 0.55 | 0.4 | 0.03 | 0.98 | 5.7 | 7,232 | 22,800 | 1.98% | 11.54% | 24% | 30,032 | 141 | 143 |
| Gerenuk | M | 31 | 0.4 | 0.1 | 0.01 | 0.51 | 3.4 | 3,764 | 13,600 | 1.65% | 10.97% | 22% | 17,364 | 224 | 248 |
| Thomson's Gazelle (N) | M+F | 21.9 | 0.25 | 0.2 | 0.01 | 0.46 | 2.5 | 3,395 | 10,000 | 2.10% | 11.42% | 25% | 13,395 | 317 | 321 |
| Thomson's Gazelle (S) | M+F | 18.6 | 0.25 | 0.15 | 0.01 | 0.41 | 1.8 | 3,026 | 7,200 | 2.20% | 9.68% | 30% | 10,226 | 374 | 420 |

Table Notes

1. For references, see notes in Table 3 of the main text.
2. Fat yield = 82%.
