# Supplementary material for: Man the Fat Hunter: The Demise of Homo erectus and the Emergence of a New Hominin Lineage in the Middle Pleistocene (ca. 400 kyr) Levant
Source: PLoS One. 2011 Dec 9;6(12):e28689. doi: 10.1371/journal.pone.0028689 (PMC3235142; doi:10.1371/journal.pone.0028689)
Supplement: Table S4 — Number of NISP per layer. (DOC) [file pone.0028689.s004.doc]

##### Table S4. Number of NISP per layer

| Body Size Group | Representative animal | Weight (kg) | Layer | | | |
| --- | --- | --- | --- | --- | --- | --- |
|  |  |  |  |  |  |  |
| **GBY** |  |  | **V-6** | **V-5** | **JB** | **II-6** |
| BSGA >1000 kg | Elephas antiquus | 6952 | 15 | 10 | 9 | 13 |
| BSGB 1000 kg | Hippopothamus amph. | 1383 | 15 | 12 | 9 | 20 |
| BSGC 250-80 kg | Oryx | 168.5 | 86 | 49 | 94 | 52 |
| BSGD 80-40 kg | Kob | 79.4 | 329 | 201 | 169 | 98 |
| BSGE 40-15 kg | Thomson's Gazelle | 21.9 | 211 | 105 | 120 | 40 |
|  |  |  |  |  |  |  |
| **Qesem** |  |  | **II** | **III** | **VI** | **V** |
| BSGA >1000 kg | Elephas antiquus | |  |  |  |  |
| BSGB 1000 kg | Buffalo | 753 | 107 | 188 | 186 | 27 |
| BSGC 250-80 kg | Oryx | 168.5 | 58 | 91 | 85 | 35 |
| BSGD 80-40 kg | Kob | 79.4 | 847 | 1009 | 1449 | 435 |
| BSGE 40-15 kg | Thomson's Gazelle | 21.9 | 0 | 0 | 0 | 0 |
